# Chinese medicine for headaches in emergency department: a retrospective analysis of real-world electronic medical records

**DOI:** 10.3389/fneur.2024.1529874

**Published:** 2025-01-20

**Authors:** Zhenhui Mao, Shirong Wu, Yuzhen Fan, Jingbo Sun, Shaohua Lyu, Qiaozhen Su

**Affiliations:** ^1^The Second Affiliated Hospital of Guangzhou University of Chinese Medicine, Guangdong Provincial Hospital of Chinese Medicine and Guangdong Provincial Academy of Chinese Medical Sciences, Guangzhou, China; ^2^The Second Clinical Medical College of Guangzhou University of Chinese Medicine, Guangzhou, China

**Keywords:** Chinese medicine, headache, emergency department, real-world study, electronic medical record

## Abstract

**Background:**

Headaches are common complaints in the emergency department (ED) and have raised concern about acute medication overuse. Chinese medicine is a major complementary and alternative medicine in China and effective for headaches. This study aims to summarize characteristics of headache patients at EDs and the utilization of Chinese medicine for headache managements in EDs.

**Methods:**

The study conducted a retrospective analysis based on existing electronic medical records at EDs from four branches of Guangdong Provincial Hospital of Chinese Medicine. Only complete medical records with a first diagnosis of headache within the specified timeframe were included. Data was extracted, screened and standardized using a structured approach. Descriptive analyses and Apriori algorithm-based association rules were employed for the study.

**Results:**

A total of 3,355 medical records were analyzed, with over 86% of headaches classified as non-urgent. Approximately 97% of the patients received a general diagnosis of headaches without further classification. Hypertension was the most prevalent concomitant diagnosis, affecting 27.42% of the patients. Western medicine was prescribed to 66% of the patients for headaches and co-existing conditions, while each type of acute medication was prescribed to fewer than 10% of the patients. Conversely, over one-third of the patients utilized headache-specific patented Chinese herbal medicine products. Additionally, oral and topical Chinese herbal medicine treatments were also administered for headaches in the emergency departments.

**Conclusion:**

The majority of headaches consulting in the EDs were non-urgent and treated with various forms of Chinese medicine, alone or in conjunction of western medicine. Chinese herbal medicine may be promoted as alternatives to Western acute medications for treating benign headaches.

## 1 Introduction

Headache ranks as the fourth most common complaint in emergency departments (EDs), accounting for approximately 2 to 4% of all chief complaints (1–4). These headaches can be primary, such as migraine, tension-type headaches, and cluster headaches, or secondary to other conditions like hypertension, brain tumors, and hemorrhages (1).

Despite the lack of universally agreed-upon guidelines for headache management in EDs across countries (1, 5–7), pharmacological treatment remains a popular approach worldwide. Pharmacological treatments involve nonsteroidal anti-inflammatory drugs (NSAIDs), antiemetic drugs, triptans, and corticosteroids (3). NSAIDs, which are the most prescribed medications for headaches, have been associated with adverse effects such as gastrointestinal complications, renal disturbances, and cardiovascular events (8, 9). Conversely, opioids are still actively prescribed for headaches in EDs, despite recommendations of avoidance due to analgesic tolerance, addiction, physical dependence, migraine chronification, increased recurrence rates, and lowered pain threshold (10–14).

Complementary and alternative medicine (CAM), including Chinese medicine, is widely used by individuals with headaches, either alone or in conjunction with conventional medications (15). Chinese medicine, which encompasses Chinese herbal medicine (CHM) decoctions, patented Chinese herbal medicine products (PCHMPs), acupuncture, and other modalities, is the mainstream CAM in China. Clinical trials have established the efficacy and safety of Chinese medicine in preventing primary headaches, including migraines and tension-type headaches (16, 17). Furthermore, the approach to managing migraines with Chinese medicine in outpatient settings has been documented (18). However, there is a lack of clarity regarding prescription patterns of Chinese medicine for acute headaches in real-world ED settings, as well as the characteristics of headache patients presenting to EDs. To address these gaps, we conducted this study to elucidate the characteristics of headaches in ED settings and the utilization of Chinese medicine for headache management in real-world clinical practice.

## 2 Methods

### 2.1 Study design

The study employs a retrospective analysis utilizing existing electronic medical records (EMRs) from ED at Guangdong Provincial Hospital of Chinese Medicine (GPHCM), which is the largest tertiary hospital that provides integrated Chinese medicine and conventional therapies for patients in China (19). The study was approved by the Human Research Ethics Committee of GPHCM with a waiver of consent (BE2024-188-01).

### 2.2 Data search and screening

The EMRs with a first diagnosis of headache between 1 January 2023 and 31 December 2023 from four EDs at GPHCM were identified and retrieved by the Information Technology Department of GPHCM. The diagnosis of headache could either be general, without specifying the type or cause (as doctors may prioritize capturing the most prominent complaint, ‘headache,’ as a diagnosis), or it could be a specific type of headache such as migraine, tension-type headache, or cluster headache. EMRs with incomplete information would be excluded for further screening.

### 2.3 Data extraction

Structured data, such as triage category, age, gender, primary diagnoses and concurrent diagnoses, treatment methods, results of brain imaging including head computed tomography (CT) and magnetic resonance imaging (MRI), medicines and herbs were extracted by Zhenhui Mao and double checked by Shaohua Lyu. It should be noted that detailed headache variables like duration of headache, previous medication history for headache episodes, associated symptoms of headache and severity of headache were not consistently available in the EMRs. In addition, doses of herbs varied among patients according to individual conditions, therefore doses of herbs were not extracted or analyzed.

### 2.4 Data standardization

Chinese medicinal herbs in different forms (granule or decoction piece) or being processed in various ways were standardized as one herb name as they share similar functions, in accordance to the World Flora Oline Plant List (20). For example, *zhi gan cao* (fried *gan cao*) was standardized as *gan cao* (Glycyrrhizae Radix *et* Rhizoma) since they were not distinguished in function. Conversely, the function of *gan jiang* (dried ginger, Zingiberis Rhizoma) is distinguished from that of *sheng jiang* (fresh ginger, Zingiberis Rhizoma Recens) according to the China Pharmacopoeia (version 2020) (21), therefore they were counted separately.

### 2.5 Data analysis

IBS SPSS statistics (version 26.0, IBM Corp., Armonk, NY, United States) was employed for descriptive analyses (22). Quantitative variables were described as mean with standard deviation and compared using Student’s t-test when appropriate. While categorical variables were presented as counts and percentages and compared by Chi square test, where feasible.

Association rule construction based on the Apriori algorithm (23, 24) was conducted to identify high-frequency herb combinations, using SPSS Modeler software (version 18.0, SPSS Inc., Chicago, IL, United States).

## 3 Results

### 3.1 General characteristics

A total of 3,355 patients attending EDs for headaches at GPHCM throughout the year 2023 were included in this study. Patients’ characteristics were presented in Table 1. Female patients constituted approximately 65% of the population and were older than male patients (48.62 years vs. 43.83 years, *p* < 0.001).

**Table 1:** Characteristics of the patients and treatment pattern.

Item	Categories	Mean (SD)
Age (years)	All patients	46.91 (22.09)
	Female	48.62 (21.72)
	Male	43.83 (22.41)

Item		Categories	Number (%)
Gender		Female	2,169 (64.65)
		Male	1,186 (35.35)
Classification of headache		Headache (not specified)	3,259 (97.14)
		Migraine headache/Tension headache/ Vascular neuropathic headache	284 (8.46)
Triage category		Life threatening	5 (0.15)
		Emergent	30 (0.89)
		Urgent (prompt care, but can wait)	408 (12.16)
		Non-urgent	2,892 (86.20)
Concomitant diagnosis		Hypertension	920 (27.42)
		Coronary atherosclerotic heart disease	291 (8.67)
		Diabetes	230 (6.86)
		Brain trauma	207 (6.17)
		Cervical spondylosis	174 (5.19)
		Acute upper respiratory tract infection	172 (5.13)
		Lacunar cerebral infarction	162 (4.83)
		Dizziness	139 (4.14)
		Hyperlipidemia	127 (3.79)
		Fever	121 (3.61)
		Sleep disorders	113 (3.37)
Brain imaging (CT/MR)		Total (CT and MR)	2,223 (66.26)
		CT	2,140 (63.79)
		MR	85 (2.53)
Treatment	Western medicine		2,227 (66.38)
	Chinese medicine	Oral PCHMPs	1,749 (52.13)
		Oral CHM decoction	1,031 (30.73)
		CHM injection	674 (20.09)
		Topical application of CHM	480 (14.31)
		Acupuncture	310 (9.24)
		Ear acupressure	125 (3.73)

CHM: Chinese herbal medicine; CT: computed tomography; PCHMPs: patented Chinese herbal medicine products; MR: magnetic resonance imaging; SD: standardized deviation.

Among the patients, only 8.46% of them were diagnosed with a primary headache, like migraine, tension-type and cluster headache. Up to 97.14% of the patients were roughly diagnosed with headaches without specific classification as their first diagnoses.

During the procedure of diagnoses, brain imaging (CT or MRI) was carried out among 66.26% of the patients, where head CT was run out for 63.79% of all headache patients and identified 0.98% of the ED patients as subarachnoid hemorrhage or intracranial hemorrhage.

In terms of concomitant diagnoses, hypertension ranked as the most common concurrent condition among 27.42% (*n* = 920) of the ED attendances, followed by coronary atherosclerotic heart disease among 8.67% of the headache patients.

A four-tier triage scale was utilized in EDs to sort or prioritize the ED attendances (25, 26). Only 1.04% of the patients were categorized into life-threatening or emergent emergencies, while up to 86.20% of the patients were justified as nonurgent conditions.

### 3.2 Treatment

In ED settings, western medicine stood as the most popular treatment method among 2,227 (66.38%) patients, followed by PCHMPs (*n* = 1,749, 52.13%), and oral CHM decoction (*n* = 1,031, 30.73%). Other treatments included Chinese herbal injection, topical application of CHM, acupuncture, and ear acupressure (Table 1).

In addition, 1,670 patients were prescribed WM in combination with CHM decoctions or PCHMPs, 557 patients utilized WM alone, while 1,128 received Chinese medicine therapies without any WM.

#### 3.2.1 Western medicine

Flunarizine was the most frequently used western medicine by 314 (9.36%) patients, followed by diclofenac sodium sustained-release tablets (*n* = 261, 7.78%) and paracetamol and tramadol hydrochloride tablets (*n* = 220, 6.56%) (Table 2).

**Table 2:** Western medicine for headaches.

Common names of western medicine	Frequency of use (%)
Flunarizine hydrochloride capsules	314 (9.36)
Diclofenac sodium sustained-release tablets	261 (7.78)
Paracetamol and tramadol hydrochloride tablets	220 (6.56)
Betahistine mesylate tablets	217 (6.47)
Paracetamol pseudoephedrine hydrochloride and dextromethorphan hydrobromide tablets	188 (5.60)
Ketorolac tromethamine injection	180 (5.37)
Mecobalamin tablets	134 (3.99)
Nifedipine controlled release tablets	118 (3.52)
Urapidil hydrochloride injection	107 (3.19)

The common names of the above medications are derived from their medication instructions.

#### 3.2.2 Patented Chinese herbal medicine products

The oral PCHMPs list indicated that *Tian shu* tablet is the most frequently prescribed PCHMP to 1,237 (36.87%) patients, followed by Gastrodin injection among 566 (16.87%) patients, and *Tong tian* oral solution by 212 (6.32%) patients (Table 3).

**Table 3:** Patented Chinese herbal medicine products for headaches.

Patented Chinese herbal medicine products	Frequency of use (%)	Targeted conditions*
*Tian shu* tablet	1,237 (36.87)	Headaches
Gastrodin injection	566 (16.87)	Headaches and dizziness
*Tong tian* oral solution	212 (6.32)	Headaches
*Yun nai ting* oral solution	115 (3.43)	Dizziness
*Yuan hu zhi tong* dripping pill	92 (2.74)	Headache, stomachache, dysmenorrhea
*Xiao chai hu* granule	73 (2.18)	Coldness
*Huo xiang zheng qi* oral solution	70 (2.09)	Coldness
*Quan tian ma* capsule	68 (2.03)	Headaches

The targeted conditions of the products referred to the Chinese Pharmacopeia (2020) (21).

#### 3.2.3 Oral Chinese herbal medicine decoction

As indicated by Table 4, the most frequently used herbs as oral decoction for headaches in EDs include Glycyrrhizae Radix *et* Rhizoma (*gan cao*, *n* = 738), *Chuanxiong* Rhizoma (*chuan xiong*, *n* = 535), Pinelliae Rhizoma (*ban xia*, *n* = 392), Gastrodiae Rhizoma (*tian ma*, *n* = 383), Bupleuri Radix (*chai hu*, *n* = 378), Poria (*fu ling*, *n* = 338), Radix Angelicae Dahuricae (*bai zhi*, *n* = 337) and Saposhnikoviae Radix (*fang feng*, *n* = 305).

**Table 4:** Herbs in oral decoctions for headaches.

Scientific names*	English name	Local names in *Pinyin*	Frequency of use (%)
1. *Glycyrrhiza uralensis* Fisch. 2. *Glycyrrhiza inflata* Bat. 3. *Glycyrrhiza glabra* L.	Glycyrrhizae Radix *et* Rhizoma	*Gan cao*	738 (22.00)
*Ligusticum striatum* DC.	*Chuanxiong* Rhizoma	*Chaun xiong*	535 (15.95)
*Pinellia ternate* (Thunb.) Breit.	Pinelliae Rhizoma	*Ban xia*	392 (11.68)
*Gastrodia elata* Bl.	Gastrodiae Rhizoma	*Tian ma*	383 (11.42)
1. *Bupleurum chinense* DC. 2. *Bupleurum scorzonerifolium* Willd.	Bupleuri Radix	*Chai hu*	378 (11.27)
*Poria cocos* (Schw.) Wolf	Poria	*Fu ling*	338 (10.07)
1. *Angelica dahurica* (Fisch.ex Hoffm.) Benth.et Hook.f. 2. *Angelica dahurica* (Fisch. ex Hoffm.) Benth.et Hook.f.var.*formosana* (Boiss.) Shan et Yuan	Radix Angelicae Dahuricae	*Bai zhi*	337 (10.04)
*Saposhnikovia di*var*icata* (Turcz.) Schischk.	Saposhnikoviae Radix	*Fang feng*	305 (9.09)
*Atractylodes macrocephala* Koidz.	Atractylodis Macrocephalae Rhizoma	*Bai zhu*	297 (8.85)
*Scutellaria baicalensis* Georgi	Scutellariae Radix	*Huang qin*	263 (7.84)
*Citrus reticulata* Blanco	Citri Reticulatae Pericarpium	*Chen pi*	260 (7.75)
*Paeonia lactiflora* Pall.	Paeoniae Radix Alba	*Bai shao*	255 (7.60)
*Pueraria lobata* (Willd.) Ohwi	Puerariae Lobatae Radix	*Ge gen*	232 (6.92)
1. *Notopterygium incisum* Ting ex H. T. Chang 2. *Notopterygium franchetii* H. de Boiss.	Notopterygii Rhizoma *et* Radix	*Qiang huo*	226 (6.74)
*Schizonepeta tenuifolia* Briq.	Schizonepetae Herba	*Jing jie*	197 (5.87)
*Mentha haplocalyx* Briq.	Menthae Haplocalycis Herba	*Bo he*	192 (5.72)
*Zingiber officinale* Rosc.	Zingiberis Rhizoma Recens	*Sheng jiang*	168 (5.0)
1. *Uncaria rhynchophylla* (Miq.) Miq. ex Havil. 2. *Uncaria macrophylla* Wall. 3. *Uncaria hirsuta* Havil. 4. *Uncaria sinensis* (Oliv.) Havil. 5. *Uncaria sessilifructus* Roxb.	Uncariae Ramulus *Cum* Uncis	*Gou teng*	163 (4.86)
*Ziziphus jujuba* Mill.	Jujubae Fructus	*Da zao*	154 (4.59)

*The scientific names of the herbs were standardized according to Chinese pharmacopeia (21). *The World Flora Oline Plant List (20).

Apriori algorithm-based association rules were conducted to identify the core herb combinations. The top ten herb combinations were displayed in Table 5, as indicated, the combinations mainly involved Glycyrrhizae Radix *et* Rhizoma (*gan cao*), Notopterygii Rhizoma *et* Radix (*qiang huo*), Saposhnikoviae Radix (*fang feng*), *Chuanxiong* Rhizoma (*chuan Xiong*), *Platycodon grandiflorum* (Jacq) A.DC. (*jie geng*), Radix Angelicae Dahuricae (*bai zhi*), Schizonepetae Herba (*jing jie*), Asarum sieboldii Miq. (*xi xin*), Gastrodiae Rhizoma (*tian ma*) and Uncariae Ramulus *Cum* Uncis (*gou teng*), which are also the main ingredients of a classical formular *Chuan xiong cha tiao san* (CXCTS).

**Table 5:** Top ten core herb combinations for headaches from oral CHM decoctions.

Consequent	Antecedent (5 herbs at maximum)	Support %	Confidence %	Lift
*Gan cao*	*Qiang huo* and *Fang feng* and *Chuan xiong*	10.5	93.5	1.72
*Chuan xiong*	*Qiang huo* and *Fang feng* and *Gan cao*	10.5	93.5	2.36
*Tian ma*	*Gou teng*	12.9	92.4	3.13
*Chuan xiong*	*Xi xin*	10.6	89.0	2.25
*Gan cao*	*Jie geng*	12.3	88.9	1.64
*Gan cao*	*Fang feng* and *Bai zhi* and *Chuan xiong*	11.3	88.8	1.64
*Chuan xiong*	*Qiang huo* and *Fang feng*	11.9	88.5	2.23
*Gan cao*	*Qiang huo* and *Fang feng*	11.9	88.5	1.63
*Fang feng*	*Jing jie* and *Chuan xiong*	11.4	88.0	3.90
*Fang feng*	*Jing jie* and *Gan cao*	10.9	87.5	3.87

The herb combinations were listed in descending order of confidence.

#### 3.2.4 Topical application of Chinese herbal medicine

The top frequently used herbs as topical patch included Sinapis Semen (*jie zi*, *n* = 275), Raphani Semen (*lai fu zi*, *n* = 275) and Perillae Fructus (*zi su zi*, *n* = 269). These herbs were used in combinations. They functioned in warming meridians and expelling Wind in Chinese medicine theory (Table 6).

**Table 6:** Herbs in topical applications for headache.

Scientific names*	English name	Herb names in *Pinyin*	Frequency of use (%)
1. *Sinapis alba* L. 2. *Brassica juncea* (L.) Czern. et Coss.	Sinapis Semen	*Jie zi*	275 (8.20)
*Raphanus sativus* L.	Raphani Semen	*Fu lai zi*	275 (8.20)
*Perilla frutescens* (L.) Britt.	Perillae Fructus	*Zi su zi*	269 (8.02)
1. *Magnolia officinalis Rehd*.et Wils. 2. *Magnolia officinalis Rehd*.et Wils.var.biloba Rehd.et Wils.	Magnoliae Officinalis Cortex	*Hou po*	166 (4.95)
1. *Angelica dahurica* (Fisch.ex Hoffm.) Benth.et Hook.f. 2. *Angelica dahurica* (Fisch. ex Hoffm.) Benth.et Hook.f.var.*formosana* (Boiss.) Shan et Yuan	Radix Angelicae Dahuricae	*Bai zhi*	125 (3.73)
*Pueraria lobata* (Willd.) Ohwi	Puerariae Lobatae Radix	*Ge gen*	125 (3.73)
1. *Euodia rutaecarpa* (Juss.) Benth. 2. *Euodia rutaecarpa* (Juss.) Benth. var. officinalis (Dode) Huang 3. *Euodia urticarial* (Juss.) Benth. var. bodinieri (Dode) Huang	Euodiae Fructus	*Wu zhu yu*	121 (3.61)

*The scientific names of the herbs were standardized according to Chinese pharmacopeia (21). *The World Flora Oline Plant List (20).

## 4 Discussion

### 4.1 Summary of results

The study described characteristics of 3,355 ED attendances with headaches from four branches of a tertiary Chinese medicine hospital and summarized the regularity of Chinese medicine for headaches. The results of our study provide real-world clinicians’ experience of prescribing Chinese medicine for acute headaches, which can contribute to evidence-based medical decision-making for headaches in clinical practice and health policy making, along with other components such as clinical research evidence and patients’ preferences and values (27).

According to the results, females took a larger percentage over males, and these female patients aged elder than male patients. A previous study a reported a similar finding (28). This female predominance may be due to the fact that females are more susceptible to headaches, particularly migraines, which can be attributed to hormonal fluctuations (29, 30).

Similar to previous reports, the study again found that most headaches are benign (8, 31), as 86.20% of the patients were classified as less urgent or nonurgent according to the triage category (25, 26). However, headaches can also be secondary to serious and life-threatening conditions like hemorrhages and brain tumors. It is critical to recognize, evaluate, and appropriately manage these dangerous secondary headaches in prevention of potential long-term disability or death (31). Brain images were excessively scanned among headache patients (32–34). However, brain imaging was not recommended as a routine practice for uncomplicated headaches in USA (35). Indications for neuroimaging should be strongly based on the patient’s clinical history and a detailed physical examination (2, 36). It is critical to recognize the red flags of a secondary headache: systemic signs, neurological symptoms, sudden onset, older age at onset, progression, papilledema, positional or precipitated by Valsalva and pregnancy, in order to save unnecessary examinations and detect early signs of life-threatening conditions (37–40).

In addition, most of the diagnoses included in this study were categorized as general headaches without further precise classifications. Non-specific diagnoses were prevalent in EDs (41). However, it is hypothesized that the lack of specificity in these diagnoses may not meet the requirements of precision medicine.

The study involved a diverse range of western medications. Overall, western medicine was prescribed to two-thirds of the ED patients. However, in terms of acute medications, only one-tenth of the ED patients received NSAIDs or tramadol, which is relatively limited compared to the higher use of acute medications in other countries (32, 34). Conversely, headache-specific PCHMPs based on Chinese Pharmacopeia (21), such as *Tian shu* tablets, Gastrodin injection, and *Tong tian* oral solution, etc., were prescribed to over one-third of the headache patients. Studies have demonstrated their anti-headache, particular anti-migraine effects, from bench to bedside (42–47). Moreover, they have been recommended in clinical guideline for headaches (48).

Additionally, tailored CHM decoctions were prescribed for acute headaches in EDs, with core herb combinations being ingredients of CXCTS. CXCTS has been widely used for headaches since ancient times and is included in headache clinical guidelines (18, 48, 49). Meta-analyses of randomized controlled trials have shown that CXCTS can significantly improve headache frequency and duration, alone or in combination with western medicine (50, 51), through regulating neurotransmitter transmission, inflammation, angiogenesis, vasomotor, immunity and other pathways (52).

In addition to oral CHM treatment, topical applications of CHM have been introduced since ancient times, yet they are seldom utilized in contemporary outpatient departments (18, 53). It is noteworthy that CHM was applied topically to headache patients in EDs. However, unlike the intranasal method described in classical literature, the application of CHM patches on acupuncture points or focal areas of the headache is more prevalent in modern clinical practice (53). Additionally, the herbs commonly used in patches, such as *jie zi*, *lai fu zi* and *zi su zi*, differ significantly from those used intranasally in classical texts (53). Both laboratory and clinical evidence are necessary to support the use of topical CHM for headaches.

Based on the comparative use of acute medications and Chinese medicine in this study, we hypothesize that various forms of CHM therapies might serve as complementary and alternative methods to acute medications, potentially reducing acute medication usage and the risk of medication overuse (54). However, the hypothesis needs further verification in future clinical efficacy trials.

### 4.2 Limitations

Some inevitable limitations were identified in this study. Firstly, being a retrospective study, the study did not include quantitative assessment of the treatment effects of CHM for acute headaches in EDs. Secondly, subgroup treatment analyses based on diagnoses were not feasible as most of the diagnoses were not specifically verified. Thirdly, the study was conducted solely in four branches of a Chinese medicine hospital located in southern China, limiting the generalizability of the results due to potential geographic differences.

## 5 Conclusion

The majority of headache cases were non-urgent, and they were generally diagnosed without further classification. Although a significant portion of patients were prescribed Western medicine, acute medications were restricted in the EDs of this Chinese medicine hospital. Instead, a considerable percentage of headache patients opted for various forms of Chinese medicine, such as headache-specific PCHMPs, oral or topical applications of CHM, as alternatives to acute medications.

## Data Availability

The original contributions presented in the study are included in the article/supplementary material, further inquiries can be directed to the corresponding authors.
